# Impact of COVID-19 Vaccination on Menstrual Cycle: A Cross-Sectional Study From Karachi, Pakistan

**DOI:** 10.7759/cureus.28630

**Published:** 2022-08-31

**Authors:** Muhammad Sualeh, Muhammad Roohan Uddin, Natalia Junaid, Muneeba Khan, Anam Pario, Quratul Ain

**Affiliations:** 1 Department of Medicine, Jinnah Sindh Medical University, Karachi, PAK; 2 Department of Anatomy, Jinnah Sindh Medical University, Karachi, PAK; 3 Department of Obstetrics and Gynecology, Jinnah Postgraduate Medical Centre, Karachi, PAK

**Keywords:** karachi, menstrual problems, womens health, covid-19 vaccine side effects, covid-19 in pakistan

## Abstract

Background

The coronavirus disease 2019 (COVID-19) disease triggered a worldwide health catastrophe. To deal with this deadly situation multiple vaccines were developed and a mass immunization program started globally. However, vaccine hesitancy was seen, especially among women of reproductive age, having concerns that the vaccine might affect their menstrual cycle. This study investigated the link between COVID-19 vaccination and menstrual abnormalities. It is essential for us to understand the effects of vaccines on menstruation as menstrual distress can have effects on everyday life, and mental and reproductive health.

Methods

A cross-sectional study was performed using self-administered online forms to collect data from all over Karachi. The sample included 384 females aged 18 years and above. The data were collected from November 2021 to February 2022.

Results

Majority of the participants were aged 21 years and had a normal body mass index (BMI). Most were moderately stressed (n=245) with 146 reporting menstrual changes post-vaccination. The difference between the post-vaccine menstruation affected (n=146) and the unaffected cohort (n=238) was significant. Other factors which likely contributed to the post-vaccine menstrual changes included Perceived Stress Scale (PSS) score, strenuous physical activity, and the pre-vaccine menstrual flow.

Conclusions

Among the women vaccinated for COVID-19, strenuous physical activity and high perceived stress levels affected the menstrual cycle. There is no denying that existing data are inadequate, which is one of the grounds for vaccination apprehension, particularly among menstruating women. To minimize this hesitation, the spread of disinformation about the vaccine's influence on the menstrual cycle must be avoided. In future research and clinical trials, menstruation-related side effects should also be investigated when developing vaccines.

## Introduction

Coronavirus disease 2019 (COVID-19), caused by a contagion called severe acute respiratory syndrome coronavirus 2 (SARS-CoV-2), has infected over 470 million people with a death toll of 6.12 million [1]. Over 1.5 million cases and over 30 thousand deaths have been reported in Pakistan due to COVID-19 [2]. One probable method of SARS-CoV-2 penetrating the cells is via the angiotensin-converting enzyme-2 receptor (ACE2). As a result, organs with high ACE2 expression may be targeted by this virus [3]. ACE2 expression was also found in ovarian granulosa cells in a prior animal study [4], suggesting that SARS-CoV-2 could also target the ovaries. COVID-19 vaccines were produced after considerable research and clinical studies, and they were licensed early for emergency use due to the severity of the issue [5]. Many lives have been saved due to the COVID-19 vaccination, which has significantly reduced hospital admissions compared to the non-vaccinated population [6]. Over 118 million have been vaccinated among the Pakistani populace, which makes up about 57% of the whole population [2]. The National Immunization Management System (NIMS) of Pakistan does not actively collect information regarding changes in menstruation after vaccination, which can concern women in their reproductive years.

A regular menstrual cycle is a marker of a healthy hypothalamic-pituitary-ovarian (HPO) axis and signifies a woman's overall health and well-being. Menstrual characteristics are not constant, and they vary from month to month throughout a person's life [7]. Changes in frequency, intensity, duration, or regularity can be considered menstrual irregularities [8]. Several factors can influence menstrual patterns; coagulopathy, ovulatory dysfunction, medication use, and modifiable factors (sudden weight loss, over-exercising, obesity, psychological disorders) all are considered [9]. A crucial factor to consider that can lead to menstrual abnormalities is mental stress. According to a recent study, elevated stress and anxiety levels during the COVID-19 pandemic were also linked to menstrual cycle irregularities [10].

The connection between vaccination and menstruation problems may lead to hesitancy in getting the vaccine. Unfortunately, after-effects of the COVID-19 vaccine on the menstrual cycle were not collected in clinical trials [11,12]. Initially, experts declared "no evidence" that a link existed between vaccination and menstrual changes. Consequently, this caused anti-vaccine groups to begin to equate the likelihood of short-term menstruation disruptions with long-term reproductive problems. Politicians, religious leaders, and wellness influencers used the commonly used frame of safeguarding women to discourage vaccination [13,14]. Cultural restrictions, lack of confidence in the healthcare system, and complacency resulted in vaccine hesitancy, especially in women [15].

The present study planned to bridge the gap between the currently available information and the overlooked aspects regarding the impact of the COVID-19 vaccine on the menstrual cycle [16]. It is critical to determine the impact of vaccination on menstruation, as a literature review study has shown that menstrual distress can interfere with activities of daily living, reproductive health, and mental health [17]. The findings of this study will aid in informing healthcare practitioners and the female population about the potential impact of the COVID-19 vaccine on their menstrual cycle and adding to the overall knowledge of COVID-19 vaccines.

This article was previously presented as preprint: Sualeh M, Uddin MR, Junaid N, Khan M, Pario A: Impact of COVID-19 vaccination on the menstrual cycle: a cross-sectional study from Karachi, Pakistan. Research Square. 2022. DOI: 10.21203/rs.3.rs-1609845/v1.

## Materials and methods

Design and setting

A descriptive cross-sectional study was conducted from November 2021 to February 2022 in Karachi, the largest city in Pakistan. The study was approved by the Institutional Review Board of Jinnah Sindh Medical University (JSMU-IRB) having approval number JSMU/IRB/2021/577.

Participants

Non-probability convenience sampling technique was used for the selection of participants. The study included females vaccinated against COVID-19, residing in Karachi, aged above 18 years, menstruating, and consented to participate. Females who were pregnant or lactating, those with a history of ovarian dysfunction, and those using oral contraceptives or intrauterine contraceptive devices were excluded from the study.

Sample size

The sample size was calculated to be 384 at a confidence interval of 95%, using the total female population aged above 18 years residing in Karachi [18]. The sample size was inflated by 10% to account for the low response rate. OpenEpi version 3.01 sample size calculator was utilized for a proportion or descriptive study.

Data collection and variables

A self-administered questionnaire was designed, which consisted of demographical questions like age, height, and marital status. The questionnaire also collected information regarding participants' menstruation, such as cycle length, menstrual flow, and usually associated symptoms, before and after getting vaccinated against COVID-19. The Perceived Stress Scale (PSS), developed by Cohen et al., was used to measure perceived stress [19]. The PSS score ranges from 0 to 40, where 0-13 is considered low stress, 14-26 moderate, and 27-40 is considered high perceived stress.

A total of 413 responses were collected, and 384 were included in the final analysis. Twenty-nine improperly and incomplete responses were excluded from the analysis. In-person interviews were not appropriate for data collection as it is taboo to talk about menstruation in Pakistan's conservative majority openly. So, the data were collected by circulating questionnaires through social media platforms. Informed consent was obtained, and all ethical considerations were observed.

Statistical analysis

Data were analyzed using SPSS version 25.0 (Armonk, NY: IBM Corp.) for Windows. Continuous variables were reported as median and interquartile range (IQR) due to non-normal distribution. Categorical variables were reported as frequencies and percentages.

A composite variable for post-vaccine menstruation was formed to divide the participants into two categories; those with post-vaccine menstruation changes and those without any. These categories were based on the answers to the dichotomous "Yes" or "No" questions. These questions were regarding menstrual irregularities, which were defined as variations in the flow, intensity, duration, or regularity of a woman's period (table in the Appendices) [19].

Binary logistic regression was performed to determine whether age, body mass index (BMI), PSS score, physical activity, marital status, pre-vaccination cycle regularity, and pre-vaccination menstrual flow were associated with the likelihood of having an impact on the menstruation of vaccinated participants. The moderating role of PSS score and physical activity in this model was identified as significant. Chi-square test was used for the comparison of categorical variables. A p-value equal to or less than 0.05 was considered statistically significant.

## Results

The median age of the participants was 21 years (IQR=2). The median BMI was 20.86 (IQR=5.5) among which 97 (25.3%) participants were underweight, 222 (57.8%) were normal, 47 (12.2%) were overweight, and 18 (4.7%) participants were obese. The median Perceived Stress Scale score was 22.00 (IQR=9), with most participants being moderately stressed (n=245), followed by high stress (n=91), and lastly low stress (n=48).

A one-way chi-square test suggested a significant difference between the two affected and unaffected groups of post-vaccine menstruation composite variable (p<0.01). The binary logistic regression model was statistically significant (df=10, n=384) which is 33.17, p≤0.001, suggesting that it could distinguish between those with or without having an impact on their menstruation after vaccination. The model explained between 8.3% (Cox and Snell R-squared) and 11.3% (Nagelkerke R-squared) of the variance in the dependent variable and correctly classified 67.4% of the cases. As shown in Table 1, only the PSS score, strenuous physical activity, and normal pre-vaccination menstrual flow contributed significantly to the model (p<0.05).

**Table 1:** Characteristics of participants and post-vaccine menstruation. IQR: interquartile range

Predictors	Affected (n=146)	Unaffected (n=238)	p-Value	Odds ratio (OR)	95% confidence interval
	Median (IQR) or n (%)	Median (IQR) or n (%)			
Age	21 (3.0)	21 (2.0)	0.981	0.999	0.946-1.055
Body mass index	21.5 (6.2)	20.7 (5.3)	0.348	1.023	0.976-1.071
PSS score	24 (9.0)	21 (8.0)	0.017	1.041	1.007-1.076
Marital status					
Unmarried	131 (89.7%)	215 (90.3%)	-	-	-
Married	15 (10.3%)	23 (9.7%)	0.743	0.844	0.306-2.329
Physical activity					
None	5 (3.4%)	15 (6.3%)	0.093	-	-
Light	57 (39.0%)	95 (40.0%)	0.383	1.621	0.547-4.806
Moderate	71 (48.7%)	121 (50.8%)	0.256	1.847	0.628-5.431
Strenuous	13 (8.9%)	7 (2.9%)	0.020	5.626	1.320-23.981
Pre-vaccination cycle regularity					
No	21 (14.4%)	27 (11.3%)	-	-	-
Yes	125 (85.6%)	211 (88.7%)	0.543	0.816	0.424-1.571
Pre-vaccination menstrual flow					
Scanty	10 (6.9%)	2 (0.8%)	0.002	-	-
Normal	111 (76.0%)	218 (91.6%)	0.007	0.118	0.025-0.562
Heavy	25 (17.1%)	18 (7.6%)	0.129	0.275	0.052-1.454

For every increase in PSS score, vaccinated participants were 1.04 times more likely to have an impact on their menstruation (OR=1.04, 95% CI {1.01, 1.08}). Furthermore, participants with strenuous physical activity were 5.63 times more likely to experience post-vaccination menstrual changes (OR=5.63, 95% CI {1.32, 23.98}). In contrast, participants with normal pre-vaccination menstrual flow were 0.12 times less likely to experience any post-vaccine menstrual change (OR=0.12, 95% CI {0.02, 0.56}).

**Figure 1:**
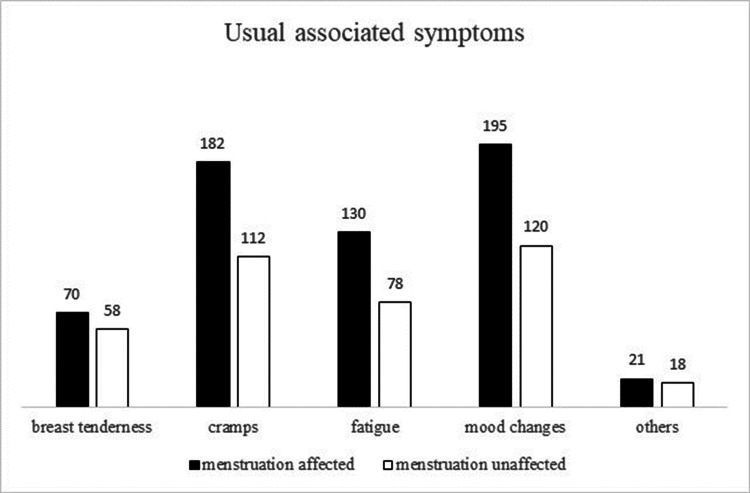
Usual associated symptoms and post-vaccine menstruation.

Mood changes were the highest reported symptom among both sub-groups (n=195 and 120), with the second being cramps (n=182 and 112), and third being fatigue (n=130 and 78). When the usual associated symptoms were compared between the two affected and unaffected subgroups of the post-vaccine menstruation composite variable, participants with usual symptoms of breast tenderness showed a statistically significant difference (p=0.04) (Figure 1).

Table 2 shows the nature of changes in menstruation post-vaccination. After taking the COVID-19 vaccine, 33 participants (21.4%) reported a decrease in their menstrual cycle length. The menstrual flow of 40 participants (15.1%) became heavier. Symptoms of 47 participants (35.3%) became much worse.

**Table 2:** Post-vaccine menstrual changes.

Variables	n (%)
In cycle duration	
Increased duration between cycles	32 (20.8%)
Decreased duration between cycles	33 (21.4%)
Disordered, no regularity	16 (10.4%)
In menstrual flow	
Gotten scantier	35 (13.2%)
Same as before getting vaccinated	10 (3.8%)
Gotten heavier	40 (15.1%)
In menstrual symptoms	
Gotten better	12 (9.0%)
Gotten worse	47 (35.3%)

## Discussion

COVID-19 vaccinations were licensed for use in the general population in various countries in late 2020 and early 2021. The rates of COVID-19 vaccine apprehension in the general population have been studied extensively worldwide and are reasonably well established [20]. According to studies, the public was hesitant to get the vaccine, particularly in South Asia, including Pakistan, resulting in individuals refusing to get the vaccine or delaying it despite its easy availability [21]. This hesitancy was more evident in women of reproductive age, as suggested by Muhaidat et al. [22]. Our study showed a change in menstrual regularity, flow, duration, and symptom intensity in more than one-third of the participants after taking the COVID-19 vaccine.

Several studies have linked high BMI with ovulatory dysfunction. Consequently, this leads to heavy menstrual bleeding, which could be because obesity is linked with high circulating estrogen levels in the blood, which is the eminent hormone of the menstrual cycle [23]. Higher BMI affects the menstrual blood flow regardless of the vaccination status of the participants, as supported by our study, which showed no relation between the two.

It was also reported that the participants who took the vaccine and practiced high-intensity physical activity were significantly more prone to menstrual abnormalities [24]. However, light and moderate physical activity showed no statistically significant effect on menstruation. Similar findings were found in the study by Kelly AK et al. which stated that athletes might experience a variety of menstruation disorders, ranging from anovulation and luteal dysfunction to oligomenorrhea and amenorrhea, all of which are linked to strenuous physical activity [25].

Furthermore, studies also showed that the COVID-19 pandemic had caused significant physical and mental disturbances, which could be due to the imposition of strict lockdowns and people being isolated from one another [26]. Women have continuously shown higher levels of stress overall [27]. The PSS scores also reported similar findings, with 70.40% of the females falling under the moderately stressed category, whereas 23.69% were highly stressed. The majority of the responses collected were from the participants of the age group 18-25 years, primarily undergraduates, making things like academic stress, lack of free time, peer competition, anxiety about academic success, and a fear of failure, significant contributors to their high-stress levels [28]. Our results reported that vaccinated individuals with high PSS scores were more likely to experience changes in their menstrual cycle which could significantly affect their personal, professional, and social achievements and activities, leading to further stress.

As of today, significant research on the subject has not been done. It remains an understudied topic; since the beginning of the immunization program for COVID-19, quite a significant fraction of people have reported experiencing sudden changes in their menstrual bleeding patterns. Individuals and groups that were hesitant to get vaccinated and anti-vaccine activists associated the risk of short-term menstrual interruptions with long-term reproductive issues [29]. Therefore, similar studies need to be conducted to clear such misconceptions and ensure that the vaccine is causing no harmful effects. In the future, clinical trials should give the same amount of consideration to menstruating women, just as pregnant and lactating women.

Limitations

The study was cross-sectional and the data were collected online, which may have resulted in a sampling bias and may not represent the entire population of all regions of Karachi. Furthermore, menstruation is still a taboo subject to address, particularly in the South Asian community, which may have contributed to respondents' reluctance to complete the questionnaire. Since it was a self-reported questionnaire, the responses' credibility remains doubtful.

## Conclusions

Our study concluded that among the women vaccinated for COVID-19, strenuous physical activity and high perceived stress levels affected the menstrual cycle. It identified that these women might experience changes in their menstrual cycle duration, flow, regularity, and associated symptoms. Such changes may impact women's daily activities, lowering their overall quality of life. In contrast, women having normal pre-vaccination menstrual flow were less likely to experience post-vaccine menstrual changes. The significant results of this study pointed more toward the association of physical activity and stress with menstrual cycle, with COVID-19 vaccine being an effect modifier. However, more studies need to be performed to inform healthcare practitioners and the female population about the possible impact that the COVID-19 vaccine can have on their menstrual cycle. In future research and clinical trials, menstruation-related side effects should also be investigated when developing vaccines.

## Appendices

**Table 3:** Self-administered demographic questionnaire. COVID-19: coronavirus disease 2019

Demographic data			
Age			
Height (in ft. inches)			
Weight (in kgs)			
Marital status		Married	
		Unmarried	
On a scale of 1-4 describe your physical activity		1 - None	
		2 - Light	
		3 - Moderate	
		4 - Strenuous	
Perceived Stress Scale; for each following question choose from the following alternatives: 0 - never, 1 - almost never, 2 - sometimes, 3 - fairly often, and 4 - very often			
l. In the last month, how often have you been upset because of something that happened unexpectedly?			
2. In the last month, how often have you felt that you were unable to control the important things in your life?			
3. In the last month, how often have you felt nervous and stressed?			
4. In the last month, how often have you felt confident about your ability to handle your personal problems?			
5. In the last month, how often have you felt that things were going your way?			
6. In the last month, how often have you found that you could not cope with all the things that you had to do?			
7. In the last month, how often have you been able to control irritations in your life?			
8. In the last month, how often have you felt that you were on top of things?			
9. In the last month, how often have you been angered because of things that happened that were outside of your control?			
10. In the last month, how often have you felt difficulties were piling up so high that you could not overcome them?			
Per-vaccination menstruation			
Did you have regular menstrual cycles before getting the COVID-19 vaccine?	Yes		
	No		
How would you describe your period flow before getting the COVID-19 vaccine?	Scanty		
	Normal		
	Heavy		
What are your usual associated symptoms during menses period? (Select all that apply)	Breast tenderness		
	Cramps		
	Fatigue		
	Mood changes, i.e., irritability, mood swings, sadness, anger, etc.		
	Other		
Post-vaccination menstruation			
Q1. Has there been a change in the regularity of your menstrual cycles after getting the COVID-19 vaccine?	Yes		
	No		
Q2. Has there been a change in your average cycle duration after getting the COVID-19 vaccine?	Yes		
	No		
Q3. If yes, then what changes have you experienced regarding cycle duration after getting the COVID-19 vaccine?	Increased duration between cycles		
	Decreased duration during cycles		
	Disordered, no regularity		
Q4. Has there been a change in your period flow after getting the COVID-19 vaccine	Yes		
	No		
Q5. If yes, then what changes have you experienced regarding period flow after getting the COVID-19 vaccine?	Gotten heavier		
	Normal/same as before getting vaccinated		
	Decreased duration during cycles		
Q6. Have you experienced any change in your usual associated symptoms during menses period after getting the COVID-19 vaccine?	Yes		
	No		
Q7. If yes, then what changes have you experienced regarding your usual associated symptoms during the menses period after getting the COVID-19 vaccine?	Gotten better		
	Gotten worse		
